# Contamination of reusable cleaning tools in healthcare settings: a study based on the longitudinal surveillance in Shanghai

**DOI:** 10.3389/fpubh.2026.1947163

**Published:** 2026-09-14

**Authors:** Xing Zhang, Lyulan Huang, Junhua Fan, Xiaofan Ji, Yilin Ge, Taiyao Chen, Jing Bi, Tingting Shi, Renyi Zhu, Liang Tian, Jian Chen

**Affiliations:** Shanghai Municipal Center for Disease Control and Prevention, Shanghai, China

**Keywords:** cleaning tools, contamination, disinfection, healthcare-associated infections, pathogen

## Abstract

**Background:**

Healthcare-associated infections (HAIs) have become global public health threat to human health. Contaminated hospital environments are increasingly recognized as critical reservoirs and vectors in the transmission of HAIs. Cleaning tools are important instruments in cleaning and disinfecting healthcare-associated facilities, of which cleanliness significantly impact the effectiveness of environmental cleaning.

**Methods:**

This research was a part of the longitudinal environmental surveillance, which was conducted in inpatient settings across Shanghai. *Staphylococcus aureus*, *Acinetobacter baumannii* and *Klebsiella pneumoniae* were collected from the sample solution. VITEK MS automatic rapid microbial mass spectrometry detection system was used to identify bacteria. The pathogen positive rate generated by three bacteria was used to indicate the contamination of cleaning tools. Pearson’s chi-square (χ^2^) test was applied for nominal categorical variables. A two-sided *p*-value < 0.05 was considered statistically significant. To identify the effects of various factors on the detection rate of pathogens, a multivariable logistic regression analysis was conducted.

**Results:**

A total of 5,000 samples of healthcare reusable cleaning tools were collected, and the positive detection rate of pathogens was 7.44%. Compared to ICU, several departments exhibited a higher risk of pathogen detection: the internal medicine department (*OR* = 1.901, *95% CI*: 1.460 ~ 2.476, *p* < 0.001), the surgical department (OR = 1.921, 95% CI: 1.443 ~ 2.556, *p* < 0.001), and other departments (*OR* = 6.183, *95% CI*: 3.264 ~ 11.711, *p* < 0.001). Regarding cleaning tools, compared to mops, buckets or basins (*OR* = 1.792, *95% CI*: 1.267 ~ 2.534, *p* = 0.001), medical trash can (*OR* = 2.631, *95% CI*: 1.911 ~ 3.623, *p* < 0.001) and other cleaning tools (*OR* = 4.490, *95% CI*: 1.055 ~ 23.127, *p* = 0.043) showed a higher risk of pathogen detection. In contrast, cleaning tools that did not receive routine disinfection were associated with a higher risk of positive detection compared to those that did (*OR* = 1.859, *95% CI*: 1.283–2.691, *p* = 0.001). There was a higher positive detection rate in medical cleaning carts disinfected by wiping with a rag than by wiping with disposable disinfectant wipes (*p* = 0.038).

**Conclusion:**

The overall pathogen positivity rate was 7.44%. Routine disinfection of cleaning tools appears to be associated with a lower detective rate of pathogens on the surface of cleaning tools. The integration of behavioral surveillance of disinfection practices, environmental microbiological surveillance, and pathogen isolate surveillance warrants consideration in future studies. Although this study provides a preliminary understanding of the contamination status and disinfection practices of cleaning tools in medical institutions, further research is warranted to evaluate specific disinfection measures and their effectiveness on these items.

## 1 Background

Healthcare-associated infections (HAIs) have become global public health threat to human health. In the healthcare system, HAIs are considered to be among the most common and serious health problems (1). HAIs are associated with increased morbidity and mortality rates, significant financial burden (2), and contribute to antimicrobial resistance (3). The rate of universal HAIs was 0.14 percent and the rate of HAIs is increasing by 0.06 percent annually (4). Although some particular healthcare environments (such as operating theatres and intensive care units) are anthropogenic environments, with the purpose of controlling HAIs, keeping the microbial contamination of the healthcare environment within accepted thresholds is necessary. Therefore, the adherence to established standards and guidelines serves as an effective strategy to reduce the incidence of these infections and implement standardized management protocols (5, 6).

Contaminated hospital environments are increasingly recognized as critical reservoirs and vectors in the transmission of HAIs (7, 8). Given that environmental surfaces are a common source of hand contamination for patients and healthcare workers alike (9), non-standard disinfection practices by staff could facilitate pathogen transmission through hand contact. Controlling the spread of pathogenic organisms in environment constitutes a key step in the management of HAIs. Enhancing environmental and facility-based cleaning and disinfection measures is recommended by most guidelines for HAIs prevention (10, 11). Reducing microbial burden in healthcare environments through effective cleaning can decrease the risk of pathogen transmission via healthcare workers’ hands or medical equipment (12). It has been suggested that HAIs could be significantly reduced by improving the cleaning and disinfection of shared medical equipment (13).

Cleaning tools are important instruments in cleaning and disinfecting healthcare-associated facilities, of which cleanliness significantly impact the effectiveness of environmental cleaning. The important role of cleaning tools in environmental sanitation has been suggested in previous study. Cleaning tools may act as reservoirs of contamination for ready-to-eat foods during preparation in retail delicatessens (14). Minimizing the contamination of cleaning tools has been considered as the part of the cleaning strategy (15). The risk of cross-contamination across surfaces and contamination of clean equipment can both increase with contaminated cleaning tools. It has been found that mops, stored wet, supported bacterial growth to very high levels and could not be adequately decontaminated by chemical disinfection (16). Emphasis should be attached to the quality of cleaning and disinfection of cleaning tools. It has been identified that contaminated cleaning tools could lead to healthcare-associated infections and further transmission in hospitals (17).

Contact isolation is recommended for patients with hospital-acquired infections (18, 19). Although contact isolation measures are various in different contexts since they are dependent on multiple factors, strengthening disinfection of the environment surrounding patients with hospital-acquired infections is critical in each case of managing HAIs. However, rather than dedicated cleaning tools, the disinfection of cleaning tools is attached importance in isolation requirements. In view of the fact that most hospitals in China are unable to provide single-room accommodations, cleaning tools are frequently shared among different areas—including the environment surrounding patients with HAIs and without HAIs, a situation that may result in cross-contamination of these cleaning tools. Notably, while disposable cleaning systems reduce environmental cross-transmission of healthcare-associated infections, reusable cleaning tools continue to pose a substantial risk of pathogen dissemination in clinical settings. Chinese standards (20) specify that, in the quality evaluation of reprocessed reusable cleaning tools, the bacteriological criterion includes the absence of detectable pathogenic microorganisms. Therefore, this study conducted a survey of cleaning supplies in healthcare facilities to assess the contamination status and current disinfecting methods, with the aim of developing strategies to improve these conditions.

## 2 Methods

### 2.1 Data resource

This research was a part of the longitudinal environmental surveillance, which was conducted in inpatient settings across Shanghai (21). A convenience sampling method was used to conduct the study. During the research period, with the exception of two districts that changed their monitoring hospitals and one district that had two monitoring sites, the remaining 13 districts of Shanghai each had one monitoring site. Ultimately, 19 hospitals were included in this study. The emergence of the COVID-19 pandemic posed unprecedented challenges to healthcare systems worldwide. During the initial phase of the outbreak, most hospitals implemented comprehensive closure protocols as part of infection control and resource reallocation strategies. Consequently, the present study experienced a temporary suspension in 2020, as data collection and on-site research activities were halted in compliance with institutional pandemic response measures. The restart of study operations was not uniform but rather occurred at time points contingent upon each institution’s specific epidemiological and operational circumstances.

### 2.2 Collection of cleaning tool samples and disinfecting information

Surface samples were collected quarterly from cleaning tools in critical departments (e.g., ICUs, surgical wards, internal medicine department, geriatrics department, respiratory medicine department, emergency department, oncology department) during the second half of 2018 to the end of 2024. Sampling was performed during routine clinical care without restricting collection to pre- or post-disinfection periods to reflect real-world microbial contamination. Concurrently, a survey on disinfection methods was conducted through a questionnaire, covering sampling subjects, timing, frequency, and disinfection techniques.

### 2.3 Strain collection and identification

Surfaces were sampled with a sterile cotton-tipped swab that was moistened with sterile phosphate-buffered saline (PBS) and rubbed vertically horizontally, and diagonally across the sampling site (100 cm^2^) delineated by a template, rotating the swab to ensure full contact of all parts of the swab tip and surface. Where the surface area of the object was less than 100 cm^2^, the entire accessible surface was sampled. For irregularly shaped objects, surface samples were collected directly using a cotton swab. *Staphylococcus aureus (SA), Acinetobacter baumannii (AB)*and *Klebsiella pneumoniae (KP)* were collected from the sample solution.

VITEK MS automatic rapid microbial mass spectrometry detection system was used to identify bacteria. The quality control strains of were *Escherichia coli* (ATCC 25922, ATCC 8739) and *Pseudomonas aeruginosa* (ATCC 27853).

### 2.4 Definitions

Based on the annual National Antimicrobial Resistance Surveillance report from the China Antimicrobial Resistance Surveillance System and expert consultation, *Staphylococcus aureus* and *Acinetobacter baumannii* were selected as the representative organisms for Gram-positive and Gram-negative bacteria, respectively, with *Klebsiella pneumoniae* subsequently added to the panel—together comprising the core species for this monitoring program. This study used the detection of *Staphylococcus aureus, Acinetobacter baumannii* and *Klebsiella pneumoniae* in cleaning tools as indicators to assess the contamination levels of cleaning tools. Since individual species-specific counts were insufficient for species-level multivariable analysis, this study combined the three types of pathogens into a single outcome measure—the pathogen positivity rate—to improve the efficiency of the testing. Accordingly, the detection of any one of the three previously mentioned bacterial types in a sample was defined as a positive result for pathogens.

The objective of this research was to assess the contamination levels of reusable cleaning supplies employed in public spaces. Notably, personal hygiene items (e.g., patient-associated basins or rags) were not included in this study. Finally, the cleaning tools included in this study comprised medical trash cans, bucket or basin, rags, mops, medical cleaning carts, and other cleaning tools. The medical trash cans were used to collect patients’ household waste or treatment-generated waste within hospital wards. The bucket or basin was used for preparing disinfectant solution or soaking rags. The rags and mops sampled in this study included those both concurrently in the wards and hung inside the cleaning and disinfection room after cleaning and disinfecting. The medical cleaning carts were mobile carts for centralized storage of disinfectants and tools such as disinfectant wipes and wet wipes, used for daily cleaning and disinfection of the environment. In this study, other cleaning tools included contaminated storage bins, cleaning supply storage bins, and the outer surfaces of wet wipe packaging. Clean supply storage bins and contaminated storage bins are designed to facilitate the proper transport, cleaning, and disinfection of clean and contaminated items, respectively.

According to China’s Hospital Classification Management Standards (22), Chinese public hospitals were divided into 3 levels (21), each further subdivided into grades. Primary hospitals are community-based healthcare centers that deliver preventive care, medical treatment, health maintenance, and rehabilitation services to a defined population, with inpatient bed capacities ranging from 20 to 99. Secondary hospitals serve as regional medical hubs, with inpatient bed capacities ranging from 100 to 499, offering comprehensive healthcare services across multiple communities while also undertaking teaching and research responsibilities. Tertiary hospitals operate at a supra-regional level, providing advanced specialized medical services and engaging in higher education and research across broader regions, and maintaining more than 500 inpatient beds. In addition, based on the evaluation scores from Part III of the Criteria for Accreditation of the Three Levels, hospitals are classified into three grades: Grade A (requiring a score of at least 90%), Grade B (≥80%), and Grade C (≥70%). Given that this study primarily examined hospital environments for inpatients—most of whom receive care in secondary and tertiary hospitals—the research was conducted within these two tiers. Specifically, the study encompassed the following hospital classes, listed in order from lower to higher levels: Class B secondary hospitals, Class A secondary hospitals, Class B tertiary hospitals, and Class A tertiary hospitals.

Wipe with a rag is defined as the application of a pre-soaked and wrung-out cloth to the target surface. Wipe with disposable disinfectant wipes is defined as the use of disposable disinfectant-impregnated non-woven wipes for surface decontamination. Immersion disinfection is defined as the submersion of objects in a freshly prepared disinfectant solution for the purpose of disinfection. High-temperature disinfection was defined as treatment in automated cleaning-disinfection equipment at 80 °C for a minimum of 10 min.

### 2.5 Statistical analysis

The detection rate of pathogens in cleaning tool samples was compared across different variables. Pearson’s chi-square (χ^2^) test was applied for nominal categorical variables. A two-sided *p*-value < 0.05 was considered statistically significant. To identify the effects of various factors on the detection rate of pathogens, a multivariable logistic regression analysis was conducted, incorporating the following variables: degree of hospital, department, sampling subjects, and routine disinfection practices. All analyses were performed using SPSS (version 25.0).

## 3 Results

### 3.1 Baseline

From 2018 to 2024, a total of 5,000 samples of healthcare cleaning tools were collected. Among these samples, 2,018, 2,002, and 980 were tested for SA, AB, and KP, respectively. And there were 94 samples tested positive for SA, 225 for AB, and 53 for KP. As a result, the overall pathogen positivity rate was 7.44%, with positivity rates of 4.88% for SA, 12.64% for AB, and 5.70% for KP on the surfaces of healthcare cleaning tools, respectively.

### 3.2 Distribution of pathogen-positive rates among different subjects

The distribution of pathogen positivity rates by hospital level, department, sampling subject, and routine disinfection practice is presented in Table 1. Positivity rates ranged from 1.96% (Class B secondary hospitals) to 8.43% (Class A secondary hospitals) across hospital levels, and from 5.53% (ICU) to 30.61% (other departments) across departments. Notably, cleaning tools subjected to daily disinfection showed a markedly lower positivity rate (7.03%) than those without (14.39%). Among cleaning tool types, trash cans (11.83%) and other cleaning tools (15.38%) exhibited the highest rates, whereas mops (4.92%), medical cleaning carts (4.34%), and rags (5.02%) showed relatively lower positivity. It was indicated that all differences in pathogen positivity rates among the aforementioned variables were statistically significant (*p* < 0.05).

**Table 1:** The distribution of positive rate for pathogens among different variables.

Variables	Negative	Positive	Positive rate	Statistical value	*p* value
Degree of hospital				χ^2^ = 10.454	*p*<0.001
Class A tertiary hospitals	1,380	91	6.19%		
Class B tertiary hospitals	2,116	184	8.00%		
Class A secondary hospitals	1,032	95	8.43%		
Class B secondary hospitals	100	2	1.96%		
Department				χ^2^ = 74.631	*p*<0.001
Internal medicine department	941	103	9.87%		
Surgical department	666	79	10.60%		
ICU	2,987	175	5.53%		
Other departments*	34	15	30.61%		
Daily disinfection or not				χ^2^ = 20.638	*p*<0.001
Non daily disinfection	238	40	14.39%		
Daily disinfection	4,390	332	7.03%		
Sample objects					
Trash cans	1,215	163	11.83%	χ^2^ = 67.520	*p*<0.001
Buckets or basins	1,078	93	7.94%		
Mops	1,083	56	4.92%		
Medical cleaning carts	1,014	46	4.34%		
Rags	227	12	5.02%		
Other cleaning tools*	11	2	15.38%		

*Other departments included geriatrics department, respiratory medicine department, emergency department, oncology department. Other cleaning tools included contaminated storage bins, cleaning supply storage bins, and the outer surfaces of wet wipe packaging.

### 3.3 The results of multivariate logistic regression analysis

As it was shown in Table 2, different factors exert varying influences on the positivity rates of pathogens. Compared to Class A tertiary hospitals, the following hospital classes showed significantly higher risks of pathogen detection: Class A secondary hospitals (OR = 1.323, 95%CI:1.003 ~ 1.745, *p* = 0.048) and Class B (OR = 1.447, 95% CI: 1.064 ~ 1.967, *p* = 0.018). Compared to ICU, several departments exhibited a higher risk of pathogen detection: the internal medicine department (OR = 1.901, 95% CI: 1.460 ~ 2.476, *p* < 0.001), the surgical department (OR = 1.921, 95% CI: 1.443 ~ 2.556, *p* < 0.001), and other departments (OR = 6.183, 95% CI: 3.264 ~ 11.711, *p* < 0.001). Regarding cleaning tools, compared to mops, buckets or basins (OR = 1.792, 95% CI: 1.267 ~ 2.534, *p* = 0.001), medical trash can (OR = 2.631, 95% CI: 1.911 ~ 3.623, *p* < 0.001) and other cleaning tools (OR = 4.490, 95% CI: 1.055 ~ 23.127, *p* = 0.043) showed a higher risk of pathogen detection. Furthermore, cleaning tools that were not subjected to routine disinfection posed a higher risk of positive detection compared to those that were disinfected daily (OR = 1.859, 95% CI: 1.283 ~ 2.691, *p* = 0.001).

**Table 2:** The results of multivariate logistic regression analysis.

Variables	*b*	Sb	Wald χ^2^	*P*	OR	95%CI
Class A tertiary hospitals					1	
Class B tertiary hospitals	0.280	0.141	3.920	0.048	1.323	1.003 ~ 1.745
Class A secondary hospitals	0.369	0.157	5.551	0.018	1.447	1.064 ~ 1.967
ICU					1	
Internal medicine department	0.642	0.135	22.718	<0.001	1.901	1.460 ~ 2.476
Surgical department	0.653	0.146	20.049	<0.001	1.921	1.443 ~ 2.556
Other departments	1.822	0.326	31.251	<0.001	6.183	3.264 ~ 11.711
Mops					1	
Buckets or basins	0.583	0.177	10.863	0.001	1.792	1.267 ~ 2.534
Medical trash can	0.967	0.163	35.120	<0.001	2.631	1.911 ~ 3.623
Other cleaning tools	1.597	0.788	4.114	0.043	4.490	1.055 ~ 23.127
Daily disinfection					1	
Non daily disinfection	0.620	0.189	10.763	0.001	1.859	1.283 ~ 2.691

### 3.4 Distribution and differences in pathogen positive detection rates of different cleaning tools surfaces under various disinfection methods

This study further analyzed the effects of different disinfection methods on various cleaning tools. The mops were disinfected by immersing in disinfectants, and the positive detection rate of them was 3.93%. With the exception of the mops, the positive rates for pathogens associated with the remaining cleaning tools are summarized in Table 3. The positive detection rates of medical trash disinfected by wiping with a rag, wiping with disposable disinfectant wipes, and immersing in disinfectant solution were 11.88, 12.90, and 5.13%, respectively. The positive detection rates of rags disinfected by high temperature and immersing in disinfectant solution were 2.42 and 5.48%. As for the bucket or basin, the positive detection rate of those disinfected by wiping with a rag and immersing in disinfectant solution was 6.92 and 7.61%, respectively, while there was no pathogen detected in the bucket or basin disinfected by wiping with disposable disinfectant wipes. The positive detection rates of medical cleaning carts disinfected by wiping with a rag, wiping with disposable disinfectant wipes, and immersing in disinfectant solution were 5.19, 0.90, and 1.19%, respectively. The positive detective rate of other cleaning tools disinfected by wiping with disposable disinfectant wipes was 25.00%, while there was no pathogen detected in other cleaning tools disinfected by wiping with a rag. Apart from the fact that there were statistically significant differences in the detection rates of pathogens on the medical cleaning carts under different disinfection methods, the effects of other disinfection methods on pathogen detection rates of different cleaning tools were not statistically significant. Specifically, there was a higher positive detection rate in medical cleaning carts disinfected by wiping with a rag than by wiping with disposable disinfectant wipes (*p* = 0.038).

**Table 3:** The distribution of pathogen-positive rate among cleaning tools by different disinfecting methods.

Cleaning tools	Disinfecting method	Negative	Positive	Positive rate	Statistical value	*P* value
Medical trash can	Wipe with a rag	942	127	11.88%	***χ***^***2***^ = 5.028	*p* = 0.081
	Wipe with disposable disinfectant wipes	81	12	12.90%		
	Immersed in disinfectant solution	111	6	5.13%		
Bucket or basin	Wipe with a rag	592	44	6.92%	***χ***^***2***^ = 0.978	*p* = 0.613
	Wipe with disposable disinfectant wipes	10	0	0.00%		
	Immersed in disinfectant solution	449	37	7.61%		
Medical cleaning carts	Wipe with a rag	803	44	5.19%	***χ***^***2***^ = 6.539	*p* = 0.038
	Wipe with disposable disinfectant wipes	110	1	0.90%		
	Immersed in disinfectant solution	83	1	1.19%		
Rags	High temperature	202	5	2.42%	***χ***^***2***^ = 3.358	*p* = 0.067
	Immersed in disinfectant solution	776	45	5.48%		
Other cleaning tools	Wipe with a rag	8	0	0.00%	***χ***^***2***^ = 2.182	*p* = 0.140
	Wipe with disposable disinfectant wipes	3	1	25.00%		

## 4 Discussion

There are multiple factors were taken accounted into the classification of Chinese hospital, including hospital’s function role, number of beds, technical capabilities, and equipment resources (22). Compared with Grade A tertiary hospitals—which have the largest bed capacity, the highest level of care, and the most comprehensive infection control systems—Grade A secondary hospitals and Grade B tertiary hospitals showed a significantly increased risk of positive pathogen detection. This may be because Grade A tertiary hospitals have more comprehensive infection control systems and procedures (23), whereas Grade A secondary hospitals and Grade B tertiary hospitals, which have a higher volume of patients, often have weaker infection control systems that make it easy to overlook the cleaning and disinfection of cleaning tools within the healthcare facility.

As reported in previous studies, the surgical department (24) exhibited a higher level of contamination than other departments, which was consistent with this study. The potential reason for the higher risk of pathogen-positive detection in the surgical and internal medicine departments may be attributed to more complex and frequent staff turnover, compared with the ICU, where personnel movement is subject to stricter restrictions. Moreover, the comparatively low contamination observed on cleaning tools in the ICU may reflect the unit’s design features. As previously demonstrated, such design can reduce exogenous microbial influx into the ICU environment (25). Conversely, other wards—including geriatric, and emergency departments—tend to have higher patient turnover, which may complicate environmental cleaning and disinfection, potentially contributing to a higher pathogen-positivity rate on cleaning tools. In this study, the limited sample size in other departments—such as oncology and emergency medicine—precluded further statistical analysis. Accordingly, the contamination status of cleaning tools in these specific settings remains to be clarified in future investigations.

It has been demonstrated that environmental disinfection is of critical importance in mitigating the dissemination of HAIs (9). Similarly, the disinfection of cleaning tools is of comparable importance. In this study, cleaning tools that were not subjected to routine disinfection in healthcare settings associated with the higher risk of positive detection of pathogens. The specific contribution of routine disinfection to reducing contamination on cleaning tools merits further investigation.

Notably, containers associated with cleaning tools—specifically, buckets or basins used for disinfectant solution and medical trash cans—showed a higher risk of pathogen-positive detection compared with mops. It was indicated that the disinfection of disinfectant-preparation containers may be a neglected aspect of routine disinfecting practices. Besides, a gap exists in the Chinese disinfection guidelines for medical institutions regarding the appropriate management and monitoring of containers used to hold liquids (26). Given the near-daily use of buckets or basins for disinfectant preparation, it may be advisable for relevant standards or guidelines to address the disinfection management of these containers. Among all cleaning-related tools, medical trash cans which used for collecting medical waste during routine medical care, exhibited the highest pathogen-positive rate, highlighting the necessity of prioritizing their cleaning and disinfection and deserves more attention in routine disinfection. The lack of a significant difference between immersion and wiping disinfection should be interpreted with caution, given the restricted sample size. This question merits re-examination in future studies with adequate statistical power. Other cleaning tools—included contaminated storage bins, cleaning supply storage bins, and the outer surfaces of wet wipe packaging—showed a higher risk of pathogen-positive detection as well. Since maintaining a clean and tidy ward environment is of great significance for preventing healthcare-associated infections since the risk of transmission rises when cleaning surfaces are restricted by spatial clutter (27), the use of supply storage bins was necessary while the disinfection of them maybe neglected in routine disinfection. In addition, more attention should be attached to the management of disposable disinfectant wipes. The contaminated outer surface could be a reservoir of pathogens and lead to HAIs through unrestricted hand hygiene. The outer surface of disposable disinfectant wipes demands attention in daily disinfecting practices.

Textiles were widely used in cleaning tools (e.g., mops, rags), which have been recognized as vectors for transmitting infections (28, 29). The cleaning and disinfection of textiles have long posed a persistent challenge in healthcare settings and continue to represent a critical focus of hospital infection control efforts (30). In the present study, pathogenic bacteria were detected on rags and mops, which were sampled during routine clinical care. Several factors may account for this observation. First, the disinfectant concentration or the contact time during the disinfection process may have failed to meet the required standards for effective microbial inactivation. Alternatively, although it has been suggested that the decontamination of rags and mops could benefit from laundering after use and drying before re-use (31), the environmental contamination existing during the hanging-drying phase could have contributed to the recontamination of these items. The precise mechanism underlying the persistence or acquisition of pathogenic bacteria following disinfection remains unclear and warrants further investigation.

Although the present study centered on reusable cleaning tools, the growing utilization of disposable alternatives in clinical disinfection protocols warrants consideration. These single-use products present an inherent advantage in infection prevention, as their application eliminates the vector for cross-contamination that is intrinsically associated with reusables. This may explain the result about the lower pathogen-positive detection in medical cleaning cart disinfected by wiping with disinfectant wipes than rags. Nevertheless, the widespread adoption of disposable sanitary products is constrained by two salient challenges that necessitate careful deliberation: the imperative of environmental sustainability and the economic constraints. With the context of significant healthcare funding gap (32), these factors collectively present a substantial barrier to the universal implementation of single-use alternatives, particularly in resource-limited healthcare settings.

As an important component of hospital disinfection practices, the contamination status and management of cleaning tools warrant due attention. Therefore, developing a rapid and efficient systematic surveillance (33) for cleaning tools is recommended to track their microbiological status. The integration of behavioral surveillance of disinfection practices, environmental microbiological surveillance, and pathogen isolate surveillance (34) merits consideration in future studies.

## 5 Conclusion

In the present study, the overall pathogen positivity rate was 7.44%. Routine disinfection of cleaning tools appears to be associated with a lower detective rate of pathogens on the surface of cleaning tools. The positive rates for pathogenic bacteria varied among different types of cleaning tools. Among all cleaning tools, medical trash cans exhibited the highest pathogen-positive rate, highlighting the necessity of prioritizing their cleaning and disinfection. The effectiveness of cleaning and disinfection for rags and mops deserves sustained attention in healthcare settings. Relevant standards or guidelines should delineate specific disinfection protocols for containers used for cleaning or disinfection purposes. The integration of behavioral surveillance of disinfection practices, environmental microbiological surveillance, and pathogen isolate surveillance warrants consideration in future studies. In addition to medical cleaning carts, which can move freely within wards, emphasis should also be placed on the contamination of other cleaning tools, such as cleaning storage bins and the outer surfaces of wet wipe packaging. Although this study provides a preliminary understanding of the contamination status and disinfection practices of cleaning tools in medical institutions, further research is warranted to evaluate specific disinfection measures and their effectiveness on these items.

## 6 Limitations

There were some limitations of this study. First of all, this study was a part of a longitudinal surveillance of health-care environment in Shanghai. Therefore, the comparability between groups was reduced because of the uneven distribution of sample sizes across the various cleaning tools and the participating wards, which were not selected through rigors study design as well. Second, the low detection rate of bacterial isolates precluded further cross-sectional comparative analysis of the distribution patterns among the three bacterial species, thereby limiting the generalizability of the findings with respect to pathogen prevalence. Thirdly, the cleaning tools in this research included both those currently in use for daily cleaning and disinfection, as well as spare cleaning tools set aside after daily cleaning and disinfection. Therefore, further study about evaluating the disinfection effectiveness of cleaning tools used in healthcare facilities should been exert in next research.

## Data Availability

Data supporting the conclusions of this article are included within the article. The datasets generated and/or analyzed during the current study are available from the corresponding author upon reasonable request.
